# Predicting Big Data Adoption in Companies With an Explanatory and Predictive Model

**DOI:** 10.3389/fpsyg.2021.651398

**Published:** 2021-04-01

**Authors:** Ángel F. Villarejo-Ramos, Juan-Pedro Cabrera-Sánchez, Juan Lara-Rubio, Francisco Liébana-Cabanillas

**Affiliations:** ^1^Department of Business Administration and Marketing, Universidad de Sevilla, Sevilla, Spain; ^2^Department of Financial Economic and Accounting, Universidad de Granada, Granada, Spain; ^3^Department of Marketing and Market Research, Universidad de Granada, Granada, Spain

**Keywords:** big data, adoption, intention to use, neural networks, predictive model

## Abstract

The purpose of this paper is to identify the factors that affect the intention to use Big Data Applications in companies. Research into Big Data usage intention and adoption is scarce and much less from the perspective of the use of these techniques in companies. That is why this research focuses on analyzing the adoption of Big Data Applications by companies. Further to a review of the literature, it is proposed to use a UTAUT model as a starting model with the update and incorporation of other variables such as resistance to use and perceived risk, and then to perform a neural network to predict this adoption. With respect to this non-parametric technique, we found that the multilayer perceptron model (MLP) for the use of Big Data Applications in companies obtains higher AUC values, and a better confusion matrix. This paper is a pioneering study using this hybrid methodology on the intention to use Big Data Applications. The result of this research has important implications for the theory and practice of adopting Big Data Applications.

## Introduction

We have been hearing the term Big Data and its benefits for some time now (McAfee and Brynjolfsson, 2012), but it is not so clear what this term means or what it encompasses. It is widely used in the field of engineering but with scarce literature on its application to business management (Verma et al., 2018), let alone from a marketing point of view.

In fact, Big Data can be grouped in two large subdivisions (Agrawal et al., 2011), one related to the generation, capture and recording of data, more related to the engineering field, and another one related to the processing and analysis of such data, which we will call Big Data Analytics (BDA).

The benefits, applications and uses that this technology can bring to companies are numerous (Wedel and Kannan, 2016; Watson, 2019), especially when it comes to making data-based decisions (McAfee and Brynjolfsson, 2012). Adopting Big Data techniques even improves users' perception of the benefits this technology can offer them (Verma et al., 2018), helping companies to innovate (Wright et al., 2019).

This adoption process is widely studied in different sectors such as healthcare (Chen et al., 2020), industrial (McMahon et al., 2020), or tourism (Yadegaridehkordi et al., 2020) although all of them refer to generic Big Data techniques while there is little literature on the adoption process of Big Data Analytics (Maroufkhani et al., 2020) as can be seen check from the appropriate literature review (Inamdar et al., 2020).

BDA may optimize many processes and improve production. Yet the real difference is in the way we process and use information for marketing management, as we will be able to improve our decision-making by enabling companies to make data-based decisions (Fan et al., 2015). Firstly, studying how to select the appropriate data sources for each marketing objective. Secondly, analyzing how to select and use the appropriate data analysis methods. Thirdly, asking how to integrate different data sources to study complex marketing problems. Fourthly, investigating how to deal with the heterogeneity of the sources. Fifthly, examining how to balance investments between different marketing intelligence techniques; and finally, implementing improvements as new, Big Data- associated technologies are developed.

In addition to all these improvements, it turns out that all the software necessary for the use and exploitation of BDA is free code, so the license prices are not an obstacle to implementing them in any type of company.

However, to implement or integrate Big Data in today's companies, a series of barriers must be overcome, such as lack of knowledge, fear of technology, resistance to change, distrust, etc. besides the limitations of the technology itself, as pointed out by Yaqoob et al. (2016).

In this paper, we aim to obtain data on the factors that affect the adoption and use of this new technology in companies, as well as to understand the possible problems for its implementation, so that we can give relevant recommendations to the professionals who make decisions. To this end, we will adapt the acceptance model of the unified theory of technology acceptance and use, UTAUT (Venkatesh et al., 2003), to which we will add inhibiting factors and other background information related to the context of Big Data adoption.

## Literature Review

Many behavioral decision theories and intention models have been developed in the scientific literature to analyze the behavior of individuals toward innovations, most of which are based on social psychology studies (Pavlou and Chai, 2002).

The adoption of a new technology is well-studied in Information Systems and psychology literature (Fishbein and Ajzen, 1975; Davis et al., 1989; Vallerand et al., 1992; Venkatesh and Davis, 2000) and its use in marketing and consumer behavior is more recent (Erevelles et al., 2016; Venkatesh et al., 2016; Wedel and Kannan, 2016).

**Figure 1:**
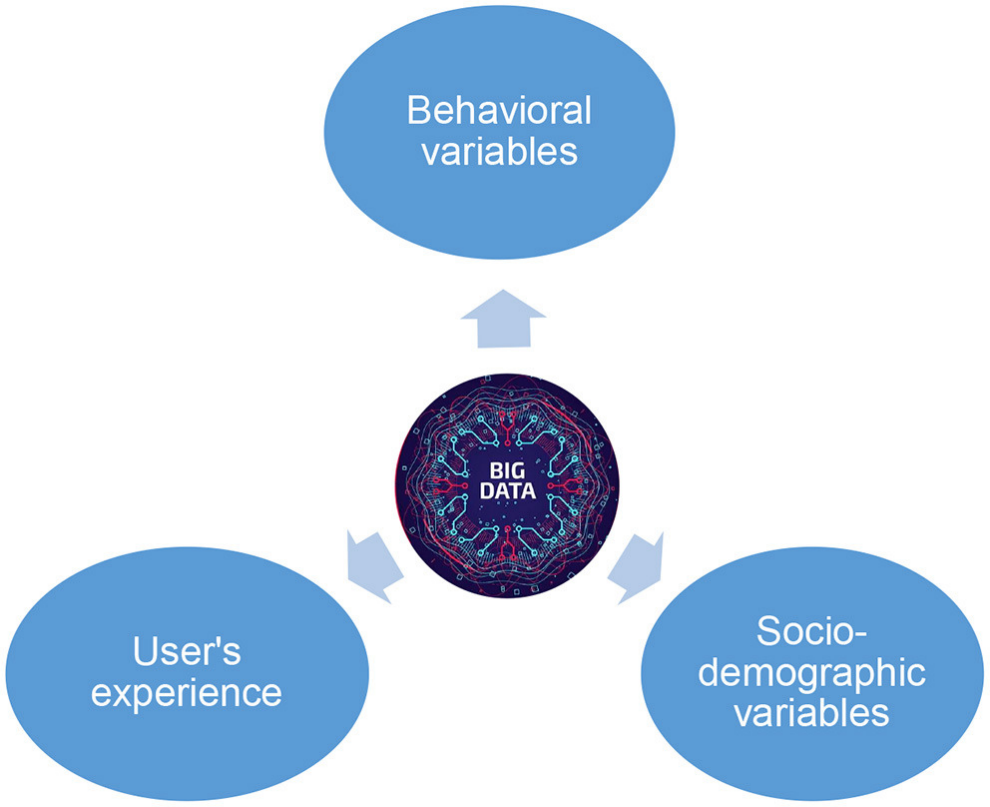
Proposed model.

**Figure 2:**
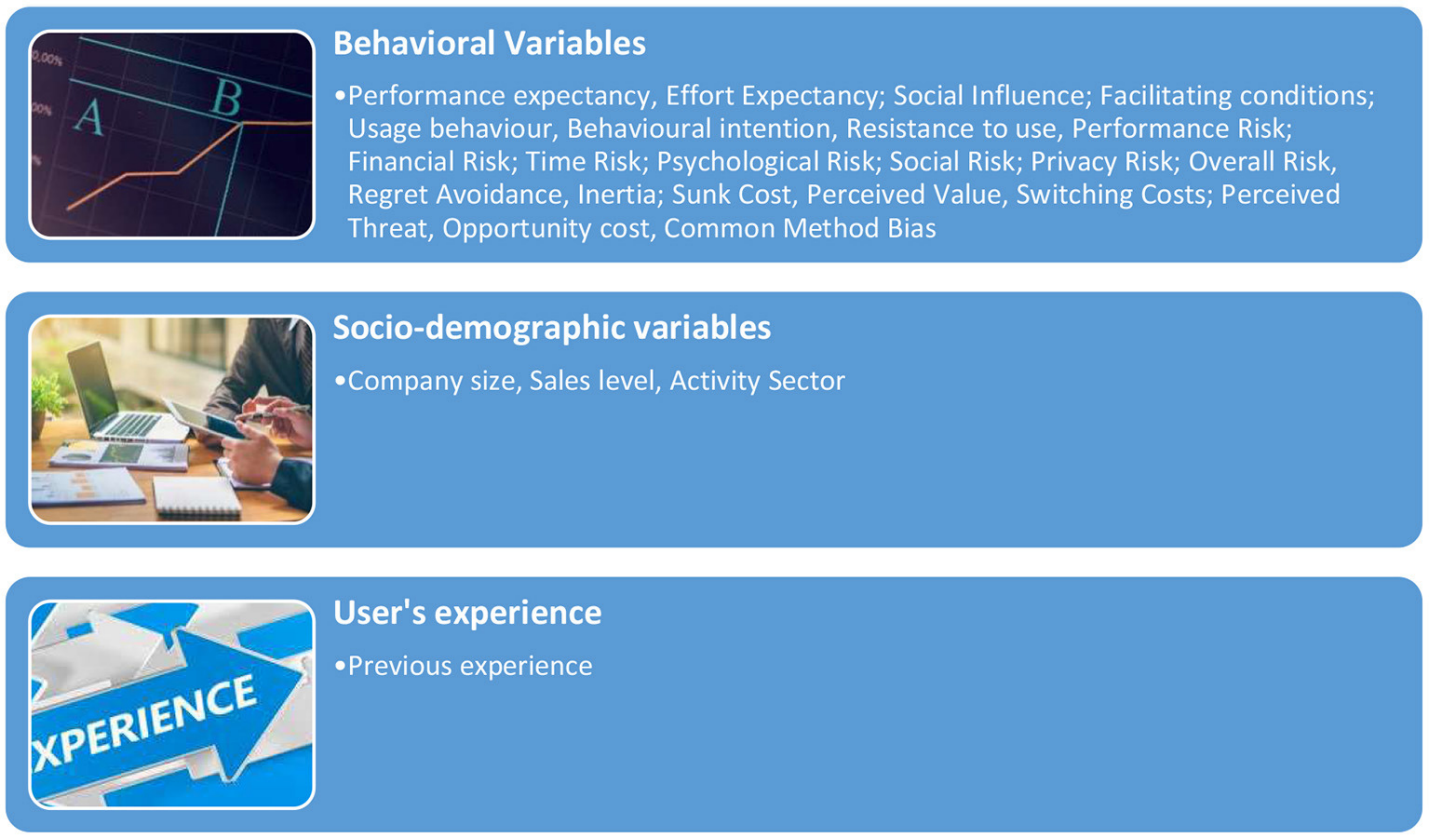
Variables analyzed.

The variables considered in this research to define intention to use Big Data system were structured in three groups: behavioral variables, socio-demographic variables, and user's experience (see Figures 1, 2).

To this end, the model chosen as a basis is the UTAUT (Venkatesh et al., 2003) since, although it is a veteran model, it is the one best suited to the adoption of technology by companies (Zhou, 2012; Al-momani et al., 2016; Arenas-Gaitán et al., 2017; Fan et al., 2018). Regarding the intention-to-use background variables from the UTAUT model, we analyzed the following:

Performance Expectancy is what we hope to achieve by applying the new technology. Its precedents lie in perceived usefulness, extrinsic motivation and fit in the job. In addition to the original study (Venkatesh et al., 2003), this construct has been used extensively in later research (Chauhan and Jaiswal, 2016; Lakhal, 2017; Cabrera-Sánchez and Villarejo-Ramos, 2019; Kalinić et al., 2019).

Effort Expectancy is the ease-of-use of the new technology, based on the precedents of perceived ease-of-use and usefulness. This construct comes from the widely used technology adoption model (TAM and Davis, 1985) as an evolution of the Perceived Ease-of-Use of that model and has been widely used in most technology adoption papers (Kim et al., 2007; Lee and Song, 2013; Chauhan and Jaiswal, 2016; Fan et al., 2018).

Social Influence is the degree to which the individual perceives that it is important for others to be using that technology. It is based on the subjective norm, social factors, and image. This construct used in the original work (Venkatesh et al., 2003), was improved in the update to UTAUT2 (Venkatesh et al., 2012) and widely used in later literature (Kim et al., 2007; Lee and Song, 2013; Duarte and Pinho, 2019).

Facilitating Conditions is the degree to which the individual believes that the company's organization and technical and human infrastructure facilitate the use of the new technology. It is based on the control of perceived behavior, facilitating conditions and compatibility. From the original paper (Venkatesh et al., 2003) its influence is ratified in the following ones (Duyck et al., 2010; Chauhan and Jaiswal, 2016; Fan et al., 2018).

Previous studies have shown that some of the UTAUT variables are losing significance, while others endow the model with greater explanatory power. Among these variables, perceived risk (Al-Saedi et al., 2020; Arfi et al., 2021) and resistance to use (Dwivedi et al., 2020; Petersen et al., 2020) are particularly worthy of note. For this reason, to extend the UTAUT and achieve a greater explanatory capacity to the Big Data adoption, we added the variables Resistance to Use (Polites and Karahanna, 2012; Lapointe and Rivard, 2017) and Perceived Risk (Featherman and Pavlou, 2003; Jia et al., 2016).

Resistance to Use, which is the negative reaction or opposition to the implementation of a new technology (Gibson, 2004). There is plenty of literature on this variable (Kim and Kankanhalli, 2009; Polites and Karahanna, 2012) even as an antecedent to the intention of use (Hsieh, 2015). Two of the main variables used to measure it are Inertia and Switching Costs as defined in the Status Quo Theory (Samuelson and Zeckhauser, 1988) and its subsequent revisions (Polites and Karahanna, 2012).

Perceived Risk is the risk perceived by the user when faced with a new technology and which acts as a brake on its implementation (Featherman and Pavlou, 2003). Perceived risk increases the predisposition to negative outcomes and thus increases resistance to using the new technology (Lapointe and Rivard, 2017). However, those who find it easier to use a new technology are those who perceive less risk in using it (Martins et al., 2014). In this paper, we have broken down the perceived risk, following the proposal of Featherman and Pavlou (2003), into: Performance Risk, Financial Risk, Time Risk, Psychological Risk, Social Risk, Privacy Risk, and Overall Risk.

In terms of consumer behavior, our review of the literature focuses on those models and theories that receive the most support specifically in marketing and information technology studies. We propose an extended model of UTAUT that includes the main variables, adapted for our research, used in previous studies on technology adoption (see Table 1).

**Table 1:** Behavioral variables and application context.

**Author(s)**	**Behavioral variables**	**Application context**
Bhattacherjee and Hikmet, 2007	Resistance to use	healthcare information technology
Featherman and Pavlou, 2003	Performance Risk; Financial Risk; Time Risk; Psychological Risk; Social Risk; Privacy Risk; Overall Risk	e-services
Tsiros and Mittal, 2000; Hsieh, 2015	Regret Avoidance	e-health services, purchase decision
Polites and Karahanna, 2012; Hsieh, 2015	Inertia; Sunk Cost	e-health services, systems used for the study
Kim and Kankanhalli, 2009; Hsieh, 2015	Perceived Value	e-health services, professional information systems
Bhattacherjee and Hikmet, 2007; Hsieh, 2015	Switching Costs; Perceived Threat	e-health services, healthcare information technology
Lu et al., 2005	Opportunity cost	Online anti-virus application

Finally, Socio-economic variables (company size, sales level, activity sector, manager level) and previous experience have been analyzed in the scientific literature (Davis, 1985; Venkatesh et al., 2016; Verma et al., 2018). This analysis has verified they have varying levels of influence on many of the relationships that determine technology adoption.

## Methodological Approach

### Study Fieldwork and Information Collection Headings

To contrast the proposed model, we devised a questionnaire and distributed it online by e-mail among managers responsible for different functional areas in Spanish companies.

To devise this questionnaire, we conducted a pre-test with five volunteer managers and as many researchers to refine it and minimize possible problems of understanding.

The data collected during the second half of 2018 and the companies with a sample of 199 participants (with response ratio of 70%), grouped by sector and turnover, is shown in Table 2.

**Table 2:** Participating companies by sales levels and activity sectors.

**Activity sector**	**<€2M**	**>€2–10M**	**>€10–43M**	**>€43M**	**(No reply)**	**Total**	**%**
Agriculture	1	3	2	1		7	3.5
Commerce and distribution	5	4	1	10		20	10.0
Construction	2		1	4		7	3,5
Education	2	1		2		5	2.5
Energy	1			3		4	2.0
Finance	1		2	8		11	5,5
Health	3			2		5	2,5
Industrial	5	3	2	6		16	8.0
Services	24	12	9	10		55	27.6
Telco	6	2	4	14	1	27	13.5
Others	10	10	6	13	2	41	20.0
(not answered)					1	1	
TOTAL	60	35	27	73	4	199	
% weight	30.1	17.5	13.5	36.0			

Based on Demuth et al. (2014) and Kordos (2016), the choice of data set size is closely related to the choice of the number of neurons in the neural network (explained in the network architecture, section Research Methodology and Experimental Design). In our case, given that the entire neural network training process is iterative, it is the network performance that indicates that we have enough data.

Specifically, the findings of the research by Vellido et al. (1999) and Yu et al. (2008) demonstrate that neural networks have a high performance in very small samples, even when the results are compared with Benchmark methods (parametric techniques). Specifically, these studies provide a broad literature review regarding the fact that network performance is related to data size.

### Variables

The dependent variable in the proposed model is a dummy variable with a value of one (1) for businessmen who have used Big Data, and zero (0) for businessmen who have not used Big Data. This variable represents the phenomenon that is explained in this research.

To explain the use of Big Data, we use many independent or explanatory variables (Table 3) that, despite having have been considered in different commercial marketing or banking marketing analyses and research, or specifically in works that investigate the adoption of other technologies, they have not yet been used as explanatory factors for the use of Big Data, which is why this research is relevant and timely.

**Table 3:** Description independent variables.

**Var**.	**Stated as**	**Source**
**PERFORMANCE EXPECTANCY (PE)**		
PE1	I think that Big Data is useful to carry out the tasks of our company	Moore and Benbasat, 1991; Venkatesh et al., 2003; McAfee and Brynjolfsson, 2012
PE2	I think that with Big Data we could do our business more quickly	
PE3	I think that with Big Data we could increase our company's productivity	
PE4	I think Big Data would improve our company's performance	
PE5	I think with Big Data you can get more information from our customers	
PE6	I think Big Data will increase the quality of information used in our company	
PE7	I think Big Data will provide valuable new information from our customers	
**EFFORT EXPECTANCY (EE)**		
EE1	Big Data would be clear and understandable to the people in our company	Venkatesh et al., 2003
EE2	It would be easy for our company to become familiar with Big Data	
EE3	It would be easy for our company to use Big Data	
EE4	I think learning Big Data would be easy for people in our company	
EE5	Generating valuable data using Big Data would be easy for our company	
**SOCIAL INFLUENCE (SI)**		
SI1	Companies that influence ours use Big Data	Venkatesh et al., 2003
SI2	The companies of reference for us use Big Data	
SI3	Companies in our environment that use Big Data are more prestigious than those that do not	
SI4	The companies in our environment that use Big Data are innovative	
SI5	Using Big Data is a status symbol in our environment	
**FACILITATING CONDITIONS (FC)**		
FC1	Our company has the necessary resources to use Big Data	Venkatesh et al., 2003
FC2	Our company has the necessary knowledge to use Big Data	
FC3	Big Data is not compatible with other systems of our company	
FC4	Our company has a person (or group of persons) available to assist with any difficulties that may arise	
**PERFORMANCE RISK (PFR)**		
PFR1	Big Data could be malfunctioning and by obtaining wrong data could lead the company to make wrong decisions	Featherman and Pavlou, 2003
PFR2	Big Data security systems are too unsafe to protect our company data	
PFR3	The probability of something going wrong with the performance of Big Data implementation is high	
PFR4	Considering the expected level of performance of Big Data, using it would be very risky for our company	
PFR5	The software associated with Big Data could malfunction and therefore provide our company with erroneous data	
**FINANCIAL RISK (FR)**		
FR1	The chances of our company losing money using Big Data are very high	Featherman and Pavlou, 2003
**TIMES RISK (TR)**		
TR1	I think that if our company uses Big Data we will waste time by having to install new type of software	Featherman and Pavlou, 2003
TR2	Using Big Data in our company would generate inconveniences since a lot of time would have to be spent solving errors	
TR3	Considering the investment in time and start-up of the System, such investment would be risky	
TR4	The probability of wasting time with system start-up and learning is very high	
**PSYCHOLOGICAL RISK (PSR)**		
PSR1	I think Big Data fits badly into our company concept	Featherman and Pavlou, 2003
PSR2	If we use Big Data, our business concept will get worse and suffer a loss of reputation	
**SOCIAL RISK (FR)**		
SR1	If we use Big Data, it will negatively affect the way others think about our company	Featherman and Pavlou, 2003
**PRIVACY RISK (PSR)**		
PR1	The probability of using Big Data and losing control of data privacy is high	Featherman and Pavlou, 2003
PR2	Using Big Data will lead to loss of privacy	
**OVERALL RISK (OR)**		
OR1	Using Big Data is globally risky	Featherman and Pavlou, 2003
OR2	It is dangerous to use Big Data	
OR3	Using Big Data exposes our company to risk	
**SUNK COST (SC)**		
SC1	A lot of time has been invested in learning how to use the current system	Polites and Karahanna, 2012; Hsieh, 2015
SC2	Much time has been invested in perfecting the skills to use the current work system	
**REGRET AVOIDANCE (RA)**		
RA1	We were wrong to choose to use Big Data	Tsiros and Mittal, 2000; Hsieh, 2015
RA2	We regret seeing the bad results that there were due to new decisions and actions made with the use of Big Data	
**INERTIA (IN)**		
IN1	We will continue to use the current data analysis method that does not include Big Data	Polites and Karahanna, 2012; Hsieh, 2015
IN2	It would be very stressful for us to switch to a new data analysis model	
IN3	We like to analyze data the way we do	
IN4	We will continue to use the current method even though we know it is not the best way to do things and that we would get more information with Big Data	
**PERCEIVED VALUE (PV)**		
PV1	Using Big Data will not increase our effectiveness at work	Kim and Kankanhalli, 2009; Hsieh, 2015
PV2	Switching to Big Data is not a good move because of the costs we might incur	
PV3	Using Big Data will not improve our efficiency	
**SWITCHING COSTS (SWC)**		
SWC1	We have already put a lot of time and effort into mastering the current way of working	Bhattacherjee and Hikmet, 2007; Hsieh, 2015
SWC2	The Big Data requires a lot of time and effort to change to this new way of working	
SWC3	Switching to Big Data Could Generate Unexpected Costs	
**PERCEIVED THREAT (PT)**		
PT1	We fear that we may lose control over the way we work if we use Big Data	Bhattacherjee and Hikmet, 2007; Hsieh, 2015
PT2	We are concerned that we may lose control over how we make decisions if we use Big Data	
**RESISTANCE TO USE (IN)**		
RU1	We do not want to use Big Data to change the way we analyze our data	Bhattacherjee and Hikmet, 2007
RU2	We do not want to use Big Data to change the way we make decisions	
RU3	We do not want to use Big Data to change the way we interact with other people in our work	
RU4	Above all, we do not want to use Big Data to change our current way of working	
**OPORTUNITY COSTS (OC)**		
OC1	I think there are alternatives to using Big Data to analyze our business data	Lu et al., 2005 (Adapted)
OC2	It would be very detrimental to our company if there was an alternative to using Big Data	
OC3	I believe that if we do not adopt Big Data, we will generate serious inconveniences to our company in the medium-long term	
**COMMON METHOD BIAS (CMB)**		
CMB1	My co-workers usually work a lot	Chin et al., 2013
CMB2	Group meetings are usually inefficient	
CMB3	It is very important to spend time with my closest family	
CMB4	University education is a good value	
**BUSINESS INFORMATION (BI)**		
BI1	Company size: (1) 0 (self-employment); (2) 1–9; (3) 10–49; (4) 50–249; (5) 250–499; (6) > 500	Venkatesh et al., 2003
BI2	Estimated annual turnover: (1) < €2 M; (2) €2 M to €10 M; (3) €10 M to €43 M; (4) > €43 M	
BI3	Sector: (1) Agriculture; (2) Commerce and distribution; (3) Telco; (4) Construction; (5) Education; (6) Energy and mining; (7) Finance; (8) Industrial; (9) Health; (10) Services; (11) Others	
BI4	Previous experience as information systems area manager: (0) No; (1) Yes	

Broadly speaking, the variables can be grouped into two large blocks, drivers, and barriers regarding the use of Big Data techniques among Spanish companies. In this respect, PE, EE, SI, FC, PV, and OC will have a positive relationship, improving the final use of these techniques by businessmen and PFR, FR, TR, PSR, SR, PR, OR, SC, RA, IN, SWC, PT, and RU will have the opposite effect, reducing their final use.

### Research Methodology and Experimental Design

#### Artificial Neural Networks Model

Artificial Neural networks (ANNs) are self-adaptive models based on computer theory and have been used in the previous literature to analyze complex non-linear relationship (Blanco et al., 2013; Kiruthika and Dilsha, 2015). To attain our objectives, we built a multilayer perceptron neural network (MLP) as a function of predictors considered as independent variables that minimizes the output or dependent variable prediction error, which is a reference procedure in the family of non-parametric models, according to Bishop (1995).

**Figure 3:**
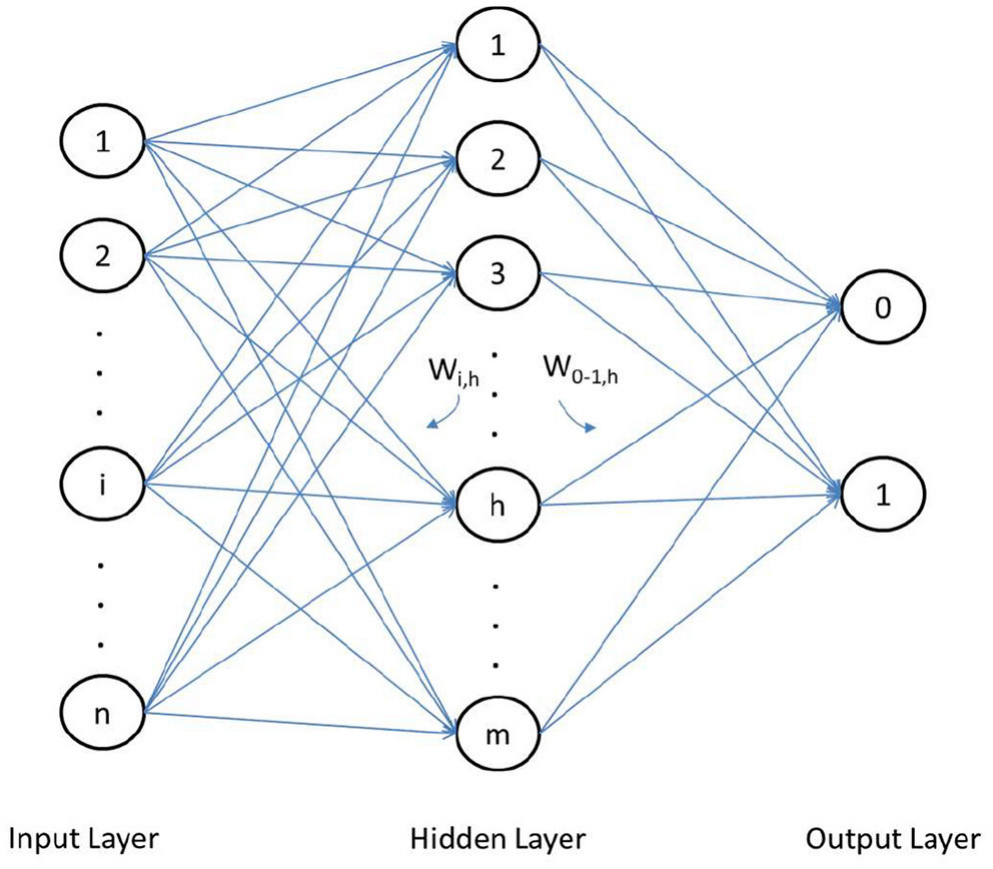
Three-layer multilayer perceptron.

Furthermore, MLP is the most used type of neural network in commercial studies (Zhang et al., 1998; Vellido et al., 1999). Based on these studies and given the characteristics of the sample, we have used the simplest building block, i.e., a three-layer perceptron (Figure 3) where the first layer has one or more neurons (nodes) representing independent (explanatory) variables, while the output layer consists of one or more neurons (nodes) which are dependent (outcome) variables, i.e., the model classification decisions. The hidden nodes in the model connect the input and output layers indirectly through a set of weights that are analogous to synaptic connections. The connections allow signals to travel through the network in parallel and in series. The synaptic weight is interpreted as the strength of the connection between the nodes (Behara et al., 2002; Garver, 2002).

The central element in the ANN (Artificial Neural Network) model is the *neural processing unit* or neuron located in the hidden layer, whose size is called H. This hidden layer is where the optimal connection weights are determined, through the learning algorithm established in the network, and among which we can distinguish {*v*_*ih*_, *i* = 0, 1, 2,., *p, h* = 1, 2,., *H*}, as the synaptic weights for the connections between p-size input and the hidden layer, and {*w*_*h*_, *h* = 0, 1, 2, ., *H*} as the synaptic weights for the connections between the hidden nodes and the output node.

The next step is to calculate the output by applying an activation function to the aggregate weighted value (West et al., 1997), where the choice of the type of activation function used in the model depends on the range of results in the output layer. In this paper we have used a sigmoid activation function calculated in a similar way to the logit function used in the logistic regression model, also used in the hidden layer of the MLP, which takes real value arguments and then transforms them into the range (0.1), according to:

$$\begin{array}{l} g\left(u\right)=\frac{e^{u}}{e^{u}+1} \end{array}$$

The output layer then contains the target (dependent) variables. In this case, the trigger function “relates” the weighted sum of units in a layer to the unit values in the correct layer, which takes a vector of real-value arguments and transforms it into a vector whose elements fall within the range (0, 1) and add up to 1.

Considering all the above, the output of the neural network from an input vector (*x*_1_, …, *x*_*p*_) is:

$$\begin{array}{l} ŷ=g\left(w_{0}+\overset{H}{\underset{h=1}{\sum }}w_{h}\;g\left(v_{0h}+\overset{p}{\underset{j=1}{\sum }}v_{ih}x_{j}\right)\right) \end{array}$$

The output of this model provides an estimate of the Big Data usage intention probability for the corresponding input vector. The final decision can be obtained by comparing this result with a threshold, usually set at 0.5, thus reaching a Big Data usage estimate, and this is the cut-off point associated with sensitivity and specificity values that are closest to one another and whose correct percentage of classification is higher.

The designed ANN continues the cross-validation procedure (West et al., 1997) consisting of the division of the sample into two subsamples. The first of these is applied to the network training, while the second is used to validate the performance of the model. This process also prevents an excess of training or over-adjustment of the neural network that would prevent the generalization of the results to the rest of the population (Garver, 2002; Deng et al., 2008).

#### Forecasting Strategy and Accuracy

An accepted criterion for assessing the explanatory and predictive quality of the ANN model is the discrimination or separation measure of 0 and 1. The discrimination and goodness-of-fit assessment measurements use the magnitudes of sensitivity, specificity, correct percentage of classification and area under the ROC curve (Dreiseitl and Ohno-Machado, 2002; Liébana-Cabanillas and Lara-Rubio, 2017). When the sensitivity values are compared to the unit difference minus the specificity 1 for different values of the threshold or cut-off point, the ROC curve to assess the performance of the ANN model is obtained.

Also, when assessing the overall predictive ability of the designed models, a priori probabilities and costs of misclassification must be considered (West, 2000). According to this author, the relative proportion of costs associated with Type I (a subject not using Big Data is misclassified as a subject using Big Data) and Type II (a subject using Big Data is misclassified as a subject not using Big Data) classification errors should be 1:5, thus highlighting the importance of measuring Type II error.

## Results and Discussion

Our empirical results are based on the information contained in the database in which, out of a total of 199 observations, 92 cases (46.23%) have used Big Data while in the remaining 107 (53.77%) Big Data has not been used for business purposes.

**Figure 4:**
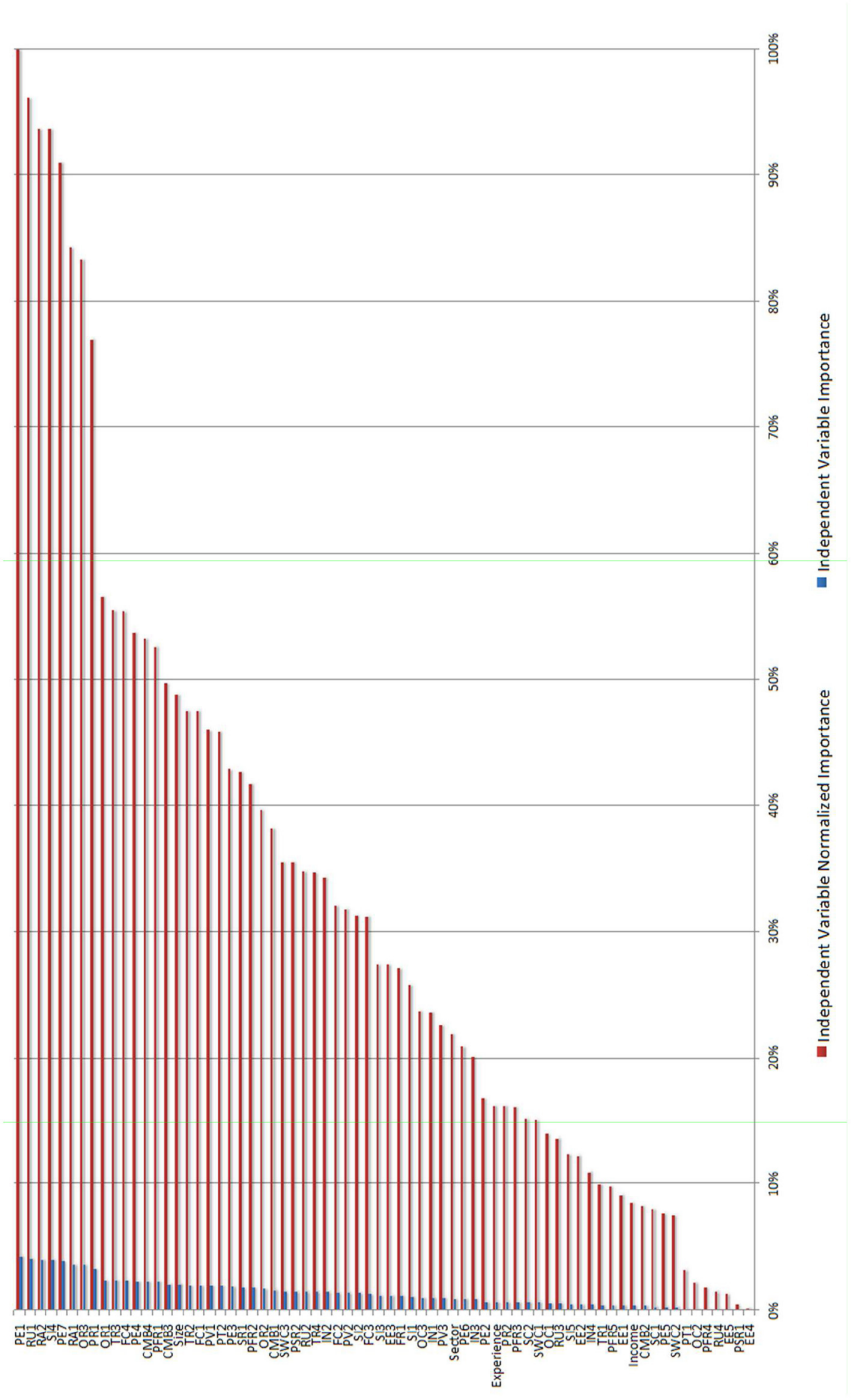
Normalized importance of the variables in MLP.

The synaptic weights obtained in our results using MLP in the prediction model learning process can be used to analyze the influence of each explanatory variable with respect to the intention of using Big Data. Figure 4 shows the overall importance and the normalized importance of each of the independent variables, showing the explanatory strength of each of the factors considered. Performs a sensitivity analysis, which computes the importance of each predictor in determining the neural network. The analysis is based on the combined training and testing samples or only on the training sample if there is no testing sample. Forteen variables present a considerable normalized importance of more than 50%, and, of these, a total of 8 variables have more than 75%.

Specifically, the variables with the greatest explanatory weight according to the designed model are: (1) Performance Expectancy, as the respondents consider that the Big Data can be useful (PE1) and that it will provide valuable customer information (PE7); (2) Resistance to use, as some businessmen do not want to change the way they analyze their data (RU1); (3) Regret avoidance due to the poor results stemming from new decisions made and actions taken with the use of Big Data (RA2 and RA1); (4) Social influence due to the use of these techniques by other companies in the respondents' environment (SI4); and finally, (5) the Overall risk (OR3) and, (6) Privacy risk (PR1) with the exposure to general and privacy risk, respectively. Consequently, drivers (1, 4) and barriers (2, 3, 5, 6) are seen to exist in the final adoption of these Big Data methodologies among the companies surveyed. These variables, which have already been used for other marketing studies, had not been considered to identify Big Data usage intention explanatory factors, and this represents an advance over previous literature. The companies in the sample consider that the acceptance and use of big data will be enhanced if they believe it improves their performance or if they see other companies in their environment using it. On the other hand, the use of big data tools may be held back by cultural and skills-related factors within the organization, as well as the perceived risks relating to its use.

As shown in Table 4, the degree of accuracy in the prediction of the constructed model is very acceptable, assuming a correct model design, because the estimates made in the training sample and in the validation sample present similar correct classification percentages. From Table 4 it can be deduced that the percentage of correctly classified subjects is 84.4%, a figure that is sustained given the good sensitivity and specificity values.

**Table 4:** Classification matrix.

**Sample**	**Observed**	**Forecast**		
		0	1	**Correct percentage**
Training	0	65	13	83.3%
	1	9	54	85.7%
	**Global percentage**	52.5%	47.5%	84.4%
Test	0	26	3	89.7%
	1	5	24	82.8%
	**Global percentage**	53.4%	46.6%	86.2%

Finally, we used the AUC that are often used in classification problems to evaluate the performance of each model (Rezáč and Rezáč, 2011). Table 5 summarizes the results, in terms of AUC, test accuracy and Type I-Type II errors of the two models tested on both the training and test samples.

**Table 5:** AUC, Type I errors, and Type II errors.

**Training sample (75%)**				**Test sample (25%)**			
**AUC**	**Test accuracy**	**Type I**	**Type II**	**AUC**	**Test accuracy**	**Type I**	**Type II**
0.823	86.37%	19.44%	23.62%	0.826	87.77%	18.96%	23.00%

In our case, it is the Type II error that quantifies false negatives that could have the greatest implications for the nature of our study. Thus, knowing that Type II error considers companies that do not use big data, but are erroneously classified as subjects that do intend to use big data, the direct implications would be an added cost derived from the study and proposal of customized products that would not materialize in the end. However, we consider our results to be within the acceptable range for this parameter (5–25%).

**Figure 5:**
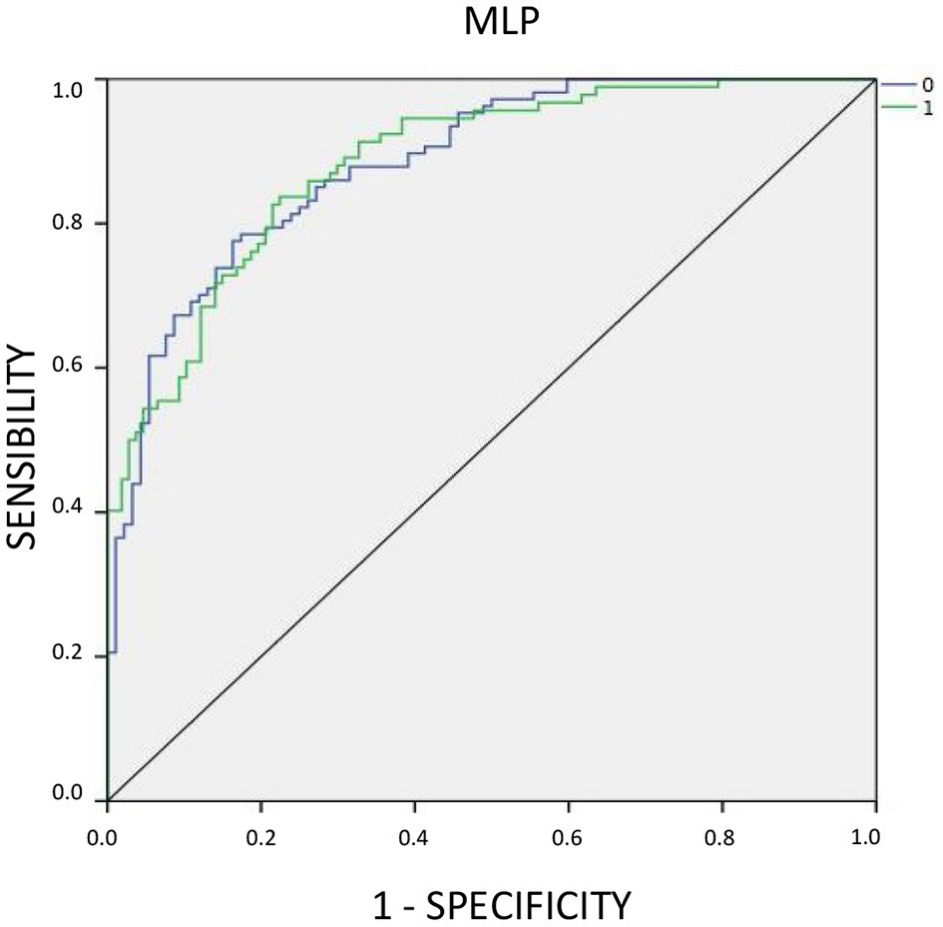
ROC Curve.

The graphical representation of this analysis is displayed in the ROC curve, which plots sensitivity and specificity values (Figure 5).

These results advance the conclusions of Featherman and Pavlou (2003), Tan et al. (2012), Venkatesh et al. (2012) and Hsieh (2015), who considered the Performance expectancy, Resistance to use, Regret avoidance, Social influence, Overall risk and Privacy risk variables as fundamental on an isolated basis in different research, but not together. Therefore, the results represent a great advance in technology adoption literature, since we have gone further in the level of analysis of the influence on the intention of use of Big Data techniques in companies. Our analysis has been conducted at the construct indicator level, determining the existence of indicators that have a greater influence such as PE1, PE7, RU1, RA1, RA2, SI4, OR3, and PR1, which we call drivers or barriers depending on whether they positively or negatively impact the intention of use. In addition, the use of predictive models leads us to conclude with a better explanatory capacity and more accurately what factors impact the adoption of this new technology.

Furthermore, these previous studies are approached in the consumer market and not from the perspective of companies making decisions on the acceptance and use of innovative technological tools.

In fact, our results indicate which variables influence the use of Big Data in companies, determining which factors act as facilitators and which are a barrier to its use. The acceptable accuracy shown in the predictive model makes us recommend that companies that want to use Big Data for information analysis take these factors into account predominantly. That is, to show the results achieved by companies that already use them to minimize the risk associated with their use and overcome the reluctance that must be faced within the organization.

## Limitations, Recommendations, and Avenues for Future Research

Despite its contributions this study is not without limitations, and these limitations provide fruitful avenues for further and future research.

Firstly, with respect to the sample used in this research, it is a limited group of companies and refers to the Spanish geographical context, preferably the service sector, which suggests that by expanding the sample and including international companies, the external reliability of the results would improve substantially and allow us to discover possible differences by country and even by sector in each country. Widening the sample may also compensate for possible bias effects do to the fact that the sample was collected before COVID-19.

Secondly, the data collection method follows a cross-sectional design, which prevents this study from analyzing how Big Data tool usage patterns evolve over time. A longitudinal design would have made it possible to test the strength of each relationship proposed, as well as to check how the results evolve once BDA is more widely implemented among the companies analyzed in the sample.

Our statistical results provide empirical evidence to support that Performance expectancy can contribute to increase the level of use of Big Data in companies, while aversion to change data processing systems contributes to reduce its probability of use. In fact, we have found evidence of an important and significant influence of other variables on Big Data usage intention.

In short, the results of the empirical study have generated interesting new knowledge for ascertaining which factors and variables businessmen perceive and value for Big Data use through the likelihood of this event occurring, providing useful information for the decision making of agents concerned about this subject. In addition, both the findings of this research and the inherent limitations represent a considerable advance over the conclusions of previous research and lay the groundwork for future research studies on companies' intention of adopting Big Data tools when faced with the challenge of using digital information in decision-making.

As follow of this research, we propose to transfer the adoption of these Big Data-based technologies in relation to their use by end users. It is true that end customers do not use Big Data techniques (at least, consciously), which is why we will use the more generic term of Artificial Intelligence applications, which do use Big Data techniques as a base (Herrera Triguero, 2014) and which could help explain the adoption of these applications in their purchase decisions or in their intention to use them.

Finally, we would like to reflect on the importance of these techniques, their relationship with the pandemic caused by COVID19 and its economic, social, and business consequences (Al Eid and Arnout, 2020). Although it is true that the proposed explanatory and predictive model could never have predicted the appearance of this disease and its consequences, we consider that it would be interesting to periodically assess the proposal update in order to verify that factors of a health nature such as the one suffered in the last year may influence the results achieved and above all, to know the possible modifications that can be proposed for the future, as well as their influence on business decision making (Abdel-Basset et al., 2021, Sharma and Gupta, 2021).

## Data Availability Statement

The raw data supporting the conclusions of this article will be made available by the authors, without undue reservation.

## Ethics Statement

Ethical approval was not provided for this study on human participants because it is not necessary. The patients/participants provided their written informed consent to participate in this study.

## Author Contributions

J-PC-S and ÁV-R: conceptualization and investigation. J-PC-S, JL-R, and ÁV-R: methodology and writing—original draft preparation. JL-R and J-PC-S: software and data curation. J-PC-S, ÁV-R, and FL-C: validation. J-PC-S, JL-R, and FL-C: formal analysis. J-PC-S, FL-C, and ÁV-R: resources. ÁV-R and FL-C: writing—review and editing. ÁV-R: supervision and project administration. All authors have read and agreed to the published version of the manuscript.

## Conflict of Interest

The authors declare that the research was conducted in the absence of any commercial or financial relationships that could be construed as a potential conflict of interest.
